# Molecular and sequencing study and identification of novel *SeM*-type in beta-hemolytic streptococci involving the upper respiratory tract in Iran

**DOI:** 10.1186/s12917-023-03772-4

**Published:** 2023-10-17

**Authors:** Sina Moghaddam, Samad Lotfollahzadeh, Taghi Zahraei Salehi, Ali Hassanpour, Hamid Tavanaei Manesh, Iraj Ashrafi Tamai

**Affiliations:** 1 Department of Internal Medicine, Faculty of Veterinary Medicine, University of Tehran, Tehran, Iran; 2 Department of Microbiology and Immunology, Faculty of Veterinary Medicine, University of Tehran, Tehran, Iran; 3 Department of Clinical Sciences, Faculty of Veterinary Medicine, Tabriz Medical Science Branch, Islamic Azad University, Tabriz, Iran

**Keywords:** Horse, Beta hemolytic streptococci, *SeM*-type, Iran

## Abstract

**Background:**

Beta-hemolytic streptococci involving the upper respiratory tract cause strangles and strangles-like diseases in horses and cause severe economic damage to the equestrian club each year. Therefore, careful epidemiological study of these bacteria, evaluation of phylogenetic connections and *SeM*-typing can be useful to determine the source and epidemiological characteristics of the disease outbreak. Isolates were analyzed using molecular and phylogenetic methods and to determine antibiotic resistance pattern in Iranian isolates. Molecular and phylogenetic methods were used to evaluate Iranian streptococcal isolates, and the similarity of the Iranian *SeM-97* sequence with other alleles was assessed using the Neighbor-joining method with the Kimura 2 Parameter statistical model. The amino acid sequence of this gene was compared with the predicted SeM-3 reference amino acid sequence (FM204883) using MEGA 7 software.

**Results:**

One type of *SeM* was found among streptococcal isolates. This type (SeM-97) was reported for the first time and was a new *SeM*. The relationship between streptococcal isolates and age, sex, race, clinical signs and geographical area was investigated. A significant relationship was observed between streptococcal isolates with age variables and clinical symptoms.

**Conclusions:**

In our study, a *Streptococcus equi subsp. equi* genotype was identified. The 97 allele of this gene has not been officially reported anywhere and is only registered in the Public databases for molecular typing and microbial genome diversity (PubMLST)-*SeM* database by Katy Webb. This was the first isolate reported and registered in the mentioned database. The isolate (Tabriz61) had the *SeM*-97 allele with clinical signs including mucopurulent discharge, abnormal sounds in lung hearing, warmth and enlargement or discharge and abscess of retropharyngeal lymph node and fever. This isolate was sensitive to penicillin, meropenem, ampicillin, cefotaxime, tetracycline, erythromycin, azithromycin, chloramphenicol, enrofloxacin and ciprofloxacin antibiotics and resistant to trimethoprim-sulfamethoxazole and gentamicin antibiotics.

**Supplementary Information:**

The online version contains supplementary material available at 10.1186/s12917-023-03772-4.

## Background

Beta-hemolytic streptococci, which involve the upper respiratory tracts, cause strangles and strangles-like disease in horses and cause severe economic damage to riding clubs yearly. Therefore, the detailed epidemiological study of these bacteria, the evaluation of phylogenetic relationships and the typing of the relevant genes can be useful for determining the origin and epidemiological characteristics of the disease outbreak [1]. In horses, pathogenic beta-hemolytic streptococci include *S. equi* causative agent of strangles, *Streptococcus equi subsp. zooepidemicus* (*S. zooepidemicus*) is an important cause of respiratory disease and metritis, and *Streptococcus dysgalactiae subsp. equisimilis* is one of the rare causes of equine lymphadenitis and placental infection and is isolated with a history of respiratory disease [2].

The main host of *S. equi* is Equidae, the agent of strangles which infects horses, ponies, donkeys and mules around the world except Iceland, and incurs great economic losses to the horse industry each year [3]. The disease has been cited in the European scientific sources in the 13th century [4]. Appropriate clinical theories were applied to strangles, and its transmission and clinical symptoms were described as early as the 17th century, even in the absence of microbiology science at that time. Strangles factor *S. equi* was first isolated by Schutz in 1888 [5]. The disease factor is an obligate parasite and needs a host for survival. Therefore, its expansion is directly related to the distribution of horse populations. Given the importance of the horse in the army in the late 1800’s, as well as its role in transportation, agriculture and leisure, strangles became a new topic for further advances in biological sciences [6]. According to the history, domestication and using horses started around 3,000 BC. Iranian tribes were among the best breeders of the horse. For this reason, throughout history, valuable horse breeds, including the Arabian horse, the Turkmen horse, and the Caspian horse, have been raised in the country. Horse breeding and horse riding are now recognized as significant economic professions worldwide, with riding holding a special place in most countries. It is considered almost as important as other sports. One of the most significant threats in horse riding clubs is strangles disease, caused by *S. equi*. It ranks as one of the most common infectious, contagious, and acute equestrian diseases globally, holding particular significance for horse health and well-being, as well as its socioeconomic impact on the industry. However, the diagnosis of *S. equi* can be challenging and prevalent among carriers. The disease can be transmitted directly and indirectly. The commune course varies from 1 to 3 weeks. It is characterized by fever, lethargy, inflammation of the mucous membranes of the upper respiratory tract, nasal infectious secretions, abscesses of the mandibular and retropharyngeal lymph nodes. After tearing, abscesses usually release a lot of puffy exudates. Young horses, typically between the ages of 1 and 5, are frequently affected, with the younger ones being more susceptible. Diagnosis is based on clinical symptoms, culture, molecular methods and serologic methods. *S. equi* is very sensitive to a wide range of antibiotics, including procaine penicillin and does not show any signs of drug resistance. Horses with high fever, severe depression, swallowing disorder and airway obstruction should be treated with antibiotics such as penicillin, chloramphenicol, erythromycin and tetracycline with nonsteroidal anti-inflammatory drugs such as phenylbutazone and flunixin meglumine [7–11]. Recent reports of *S. zooepidemicus* have been isolated from horses with a history of respiratory disease or strangles-like disease; however, infection with *Streptococcus dysgalactiae subsp. equisimilis* is rare and is considered an opportunistic infection [12]. Several epidemiological studies have shown that *S. zooepidemicus* was the main cause of purulent respiratory infections in horses and foals [13–15].

Due to the high prevalence of infection with beta-hemolytic streptococci bacteria in Iran, the present study was proposed by researchers to further evaluate the level of equestrian clubs in the northwestern provinces of Iran with a more detailed clinical and bacterial study. Molecular test was evaluated on *SeM*, *sodA*, *seeI* and *streptokinase* genes. Also, all isolated strains were evaluated for antibiotic susceptibility. The primary objective of this research was to examine cases of equine respiratory tract infections within breeding facilities situated in three provinces: West Azerbaijan, East Azerbaijan, and Ardabil. Specifically, the study sought to explore instances of these infections caused by beta-hemolytic streptococci and to analyze various risk factors associated with the disease within these regions. Remarkably, this investigation represents a pioneering effort of this magnitude in Iran.

## Results

### PCR assay for the detection and identification of Streptococcus spp.

**Fig. 1:**
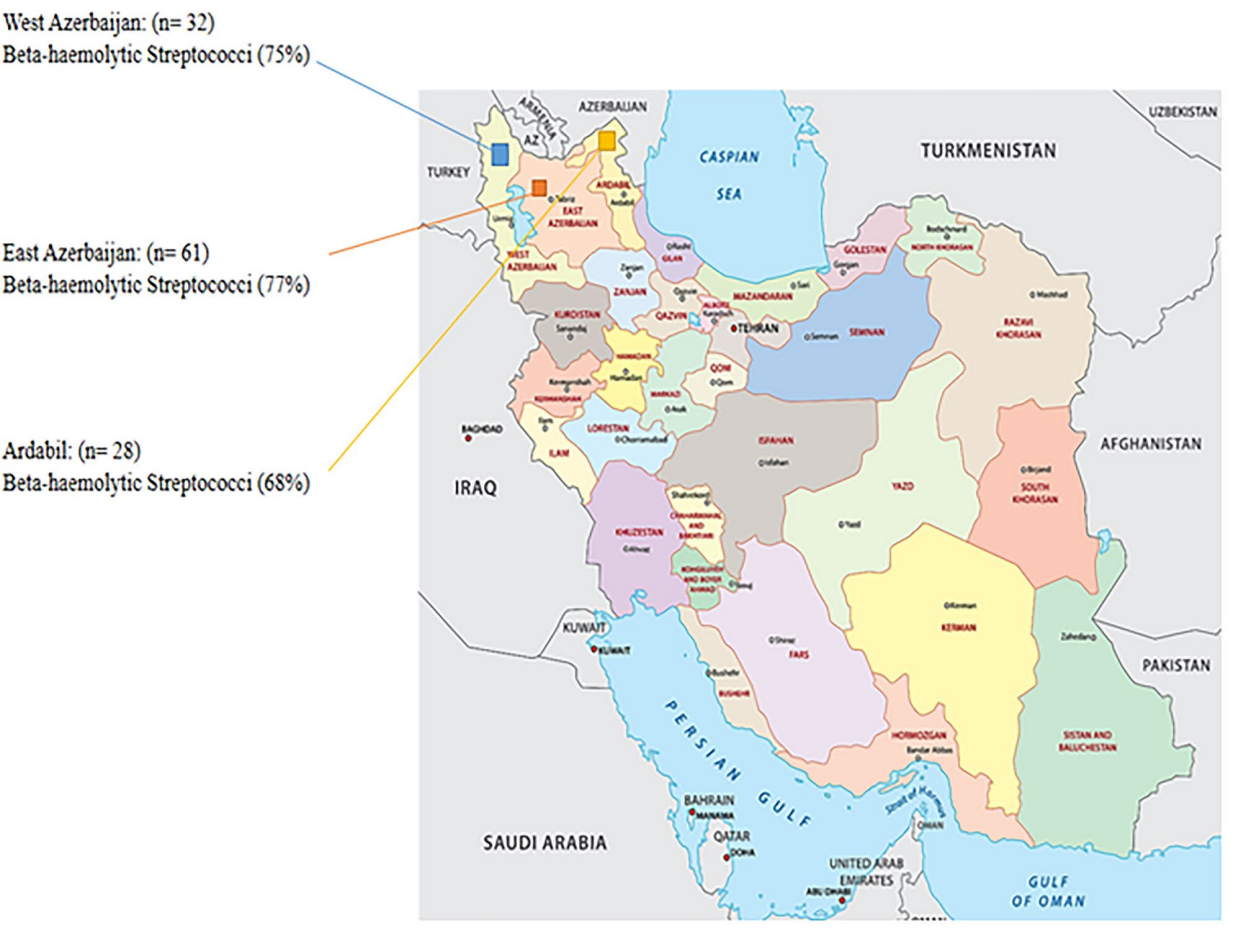
Map of the geographical distribution of the abundance of beta-hemolytic streptococci in the present study

In PCR test, 121 organisms were identified for 16 S rRNA gene and also for *SeM*, *sodA*, *SeeI* and *streptokinase* precursor genes. Table 1 displays the distribution of negative and positive cases of beta-hemolytic infections, as determined by PCR analysis, revealing a highly significant relationship (*P* < 0.001). The geographical distribution map of the abundance of beta-hemolytic streptococci in the present study is shown in Fig. 1.

**Table 1:** Absolute and relative frequency distribution (percentage) of positive and negative cases of beta-hemolytic streptococci infections based on PCR test

Group	Absolute frequency	Relative frequency	*P*-value
Negative	31	25.62	0.001
*Streptococcus equi*	1	0.82	
*Streptococcus zooepidemicus*	88	72.74	
*Streptococcus equisimilis*	1	0.82	
Total	121	100	

Electrophoresis of the final PCR product on the target genes of this study is shown in Figs. S1 to S4.

### Association and importance of risk factors in the occurrence of strangles and like-strangles

The role of host and environmental factors were assessed based on PCR. The frequency of beta-hemolytic infections in horses under 2 years of age was 87.09%, 2 to 6 years was 73.17%, 6 to 10 years was 82.6% and more than 10 years was 53.8%. Table 2 shows the distribution of the frequency of negative and positive cases by age. This table shows a significant relationship between beta-hemolytic infections and horse age (*P* < 0.05).

**Table 2:** Absolute and relative frequency distribution (percentage) of positive and negative cases of beta-hemolytic streptococci infections by age (by year)

Group	Positive		Negative		*P*-value
	Absolute f.	Relative f.	Absolute f.	Relative f.	
<2	27	87.09	4	12.91	0.026
2–6	30	73.17	11	26.83	
6–10	19	82.16	4	17.40	
>10	14	53.80	12	46.20	
Total	90	74.40	31	25.60	

The frequency of beta-hemolytic infections in stallions was 72.5% and in mares it was 78%. Table 3 shows the frequency distribution of negative and positive items by gender. There is no significant relationship between beta hemolytic infections and sex (*P* > 0.05).

**Table 3:** Absolute and relative frequency distribution (percentage) of positive and negative cases of beta-hemolytic streptococci infections by sex

Group	Positive		Negative		*P*-value
	Absolute f.	Relative f.	Absolute f.	Relative f.	
Stallion	58	72.50	22	27.50	0.508
Mare	32	78	9	22	
Total	90	74.40	31	25.60	

The frequency of beta-hemolytic infections was 80.3% in Arab, 65.1% in Kurdish, 75% in cross-breed and 77.8% in Thoroughbred horses. Table 4 shows the frequency distribution of negative and positive cases by race. There is no significant relationship between beta-hemolytic infections and race (*P* > 0.05).

**Table 4:** Absolute and relative frequency distribution (percentage) of positive and negative cases of beta-hemolytic streptococci infections by race

Group	Positive		Negative		*P*-value
	Absolute f.	Relative f.	Absolute f.	Relative f.	
Arab	49	80.30	12	19.70	0.373
Kurdish	28	65.10	15	34.90	
Cross-breed	6	75	2	25	
Thoroughbred	7	77.80	2	22.20	
Total	90	74.40	31	25.60	

Frequency of beta-hemolytic infections in horse breeding clubs in: Tabriz 82.4%, Khoy 75%, Julfa 55.6%, Marand 70%, Salmas 75%, Urmia 77.8%, Miandoab 72.7%, Ardabil 68.8%, Sarein 66.7%, Osku 87.5%. Table 5 shows the frequency distribution of negative and positive cases by geographical area. The Chi-squared test results show that there is no significant relationship between the geographical areas of the northwestern provinces of Iran and the positive and negative results of the bacterial culture test (*P* > 0.05). The highest and lowest positive results were observed in Tabriz and Salmas, respectively.

**Table 5:** Absolute and relative frequency distribution (percentage) of positive and negative cases of beta-hemolytic streptococci infections by geographical area

Group	Positive		Negative		*P*-value
	Absolute f.	Relative f.	Absolute f.	Relative f.	
Tabriz	28	82.40	6	17.60	0.887
Khoy	6	75	2	25	
Julfa	5	55.60	4	44.40	
Marand	7	70	3	30	
Salmas	3	75	1	25	
Urmia	7	77.80	2	22.20	
Miandoab	8	72.70	3	27.30	
Ardabil	11	68.80	5	31.30	
Sarein	8	66.70	4	33.30	
Osku	7	87.50	1	12.50	
Total	90	74.40	31	25.60	

In the present study, the frequency of clinical signs is shown in Table 6. Two clinical findings were excluded from the statistical analysis of the study since the clinical signs were unique. There was a significant relationship between beta-hemolytic infections and clinical signs (*P* < 0.001).

**Table 6:** Absolute and relative frequency distribution (percentage) of positive and negative cases of beta-hemolytic streptococci infections by clinical signs

Group	Positive		Negative		*P*-value
	Absolute f.	Relative f.	Absolute f.	Relative f.	
Mucopurulent discharge	30	41.09	43	58.91	<0.001
Mucopurulent discharge, abnormal sounds in the lungs hearing, warmth and enlargement or discharge and abscess of submandibular lymph node, fever	8	100	0	0	
Mucopurulent discharge, abnormal sounds in the lungs hearing, warmth and enlargement or discharge and abscess of retropharyngeal lymph node	3	100	0	0	
Mucopurulent discharge, abnormal sounds in the lungs hearing, warmth and enlargement or discharge and abscess of retropharyngeal lymph node, fever	4	100	0	0	
Mucopurulent discharge, abnormal sounds in the lungs hearing,	4	100	0	0	
Total	88	73.95	31	26.05	

Clinical signs (5-year-old Arabian mare, Tabriz) registered for one strain of *S. equi* (*SeM*- 97 strain) with access number OL332314 of NCBI database included mucopurulent discharge, abnormal sounds in lung hearing, warmth and enlargement or discharge and abscess of retropharyngeal lymph node and fever.

Also, the clinical sign (1-year-old Kurdish stallion, Sarein) registered for one strain of *Streptococcus dysgalactiae subsp. equisimilis* with access number OL332313 of the NCBI database included only mucous-purulent secretions.

The present study showed a significant relationship between the occurrence of strangles and strangles-like diseases and the age variable and clinical symptoms. The disease is more common in younger horses, and the presence of severe clinical signs will increase the likelihood of a positive PCR test result.

In beta-hemolytic streptococcal isolates, out of 90 isolates, the highest resistance to gentamicin (67.8%), trimethoprim-sulfamethoxazole (47.8%) and ciprofloxacin (25.6%) was observed. In contrast, the highest susceptibility to antibiotics was observed as: penicillin (85.6%), meropenem (82.2%), ampicillin (76.7%), cefotaxime (71.1%), tetracycline (67.8%) and erythromycin (67.8%) (Table 7).

**Table 7:** Absolute and relative frequency of antibiotic susceptibility of beta-hemolytic streptococci isolates

Antibiotics	Sensitive		Intermediate		Resistant	
	Absolute f.	Relative f.	Absolute f.	Relative f.	Absolute f.	Relative f.
Cefotaxime	64	71.10	9	10	17	18.90
Tetracycline	61	67.80	9	10	20	22.20
Trimethoprim-sulfamethoxazole	39	43.30	8	8.90	43	47.80
Meropenem	74	82.20	7	7.80	9	10
Chloramphenicol	58	64.40	16	17.80	16	17.80
Erythromycin	61	67.80	12	13.30	17	18.90
Azithromycin	54	60	16	17.80	20	22.20
Ciprofloxacin	56	62.20	11	12.20	23	25.60
Penicillin	77	85.60	6	6.70	8	7.80
Gentamicin	26	28.90	3	3.30	61	67.80
Ampicillin	69	76.70	9	10	12	13.30
Enrofloxacin	53	58.90	12	13.30	25	27.80

### Sequence analysis

**Fig. 2:**
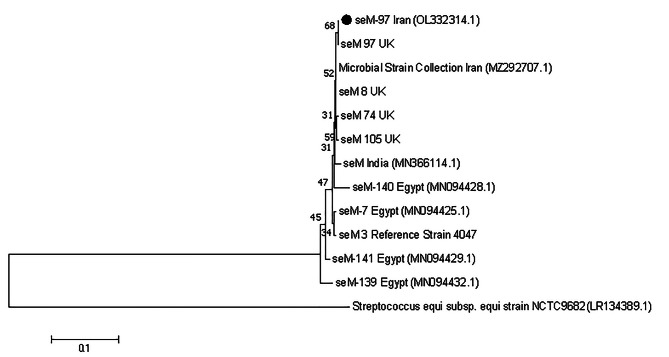
*SeM* gene cladogram by Neighbor joining method with Kimura 2 Parameter statistical model and Boot Strap 1000. The isolate from the present study is characterized by a black circle

**Fig. 3:**
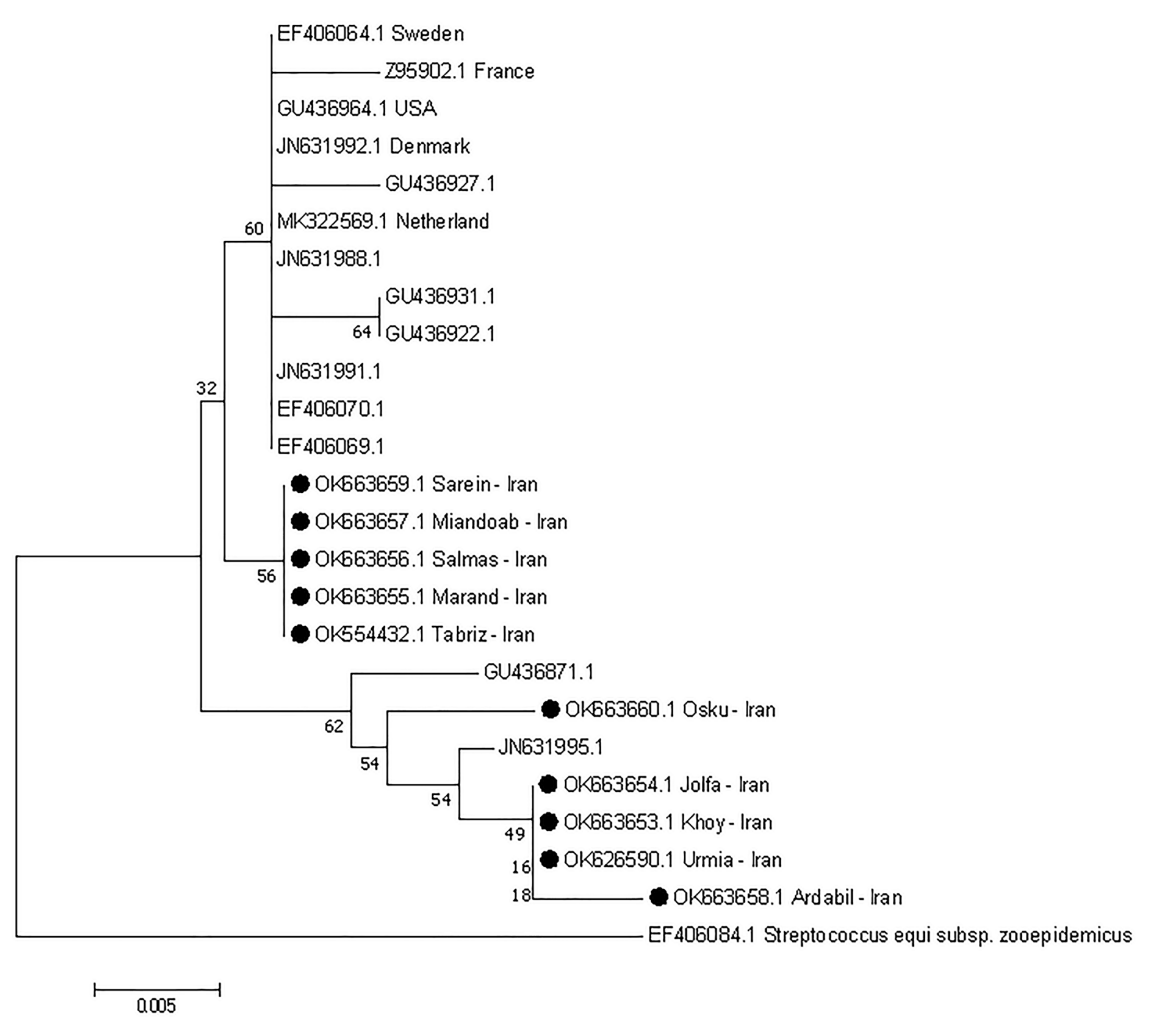
Cloudogram of *sodA* gene by Neighbor joining method with Kimura 2 Parameter statistical model and Boot Strap 1000. Isolates from the present study are marked with a black circle

In the present study, out of 121 cases of streptococci identified, only one case of allele *SeM*-97, M protein (Fig. 2) was identified and also sequencing in terms of gene *sodA* was performed in samples with severe clinical signs in each city, as shown in Fig. 3; Phylogenetic tree and the isolates of this study are indicated by a circle.

The corresponding allele has been documented in the PubMLST-SeM database, marking the initial report of the existence of this gene (*SeM-97*) within Iran.

In the new *SeM*-97 allele, four amino acid changes (related to the amino acids aspartic acid, threonine, proline and methionine) relative to *SeM*-3 of *S. equi* reference strain 4047 were identified in this study (Table 8). First, the nucleic acid sequence was converted to protein sequence by Expasy program, and the protein’s three-dimensional structure was predicted using the online software Robetta and Lometz. Subsequently, the protein structure was analyzed utilizing the jmol software, and the positions of the mutations were identified through specific commands.

**Table 8:** Comparison of the amino acid sequence from the transcript of *S. equi SeM* gene sequence with the reference strain 4047

Accession no.	Allele	Amino acid at codon No.									
		38	52	58	65	80	92	107	116	143	
FM204883	Strain 4047 (*SeM*-3)	Ser	Leu	Glu	Ala	Val	Ser	Val	Lys	Ser	
OL332314	*SeM*-97	*	*	**Asp**	**Thr**	*	**Pro**	**Met**	*	*	

## Discussion

A 2012 study by Preziuso and Cuteri used a multiplex PCR to detect and differentiate beta-hemolytic streptococci in clinical specimens. The results of differential multiplex PCR included 45 *Streptococcus dysgalactiae subsp. equisimilis*, 99 *S. zooepidemicus* and 6 *S. equi* out of 150 positive cases cultured from nasopharyngeal swabs, tracheal aspiration, guttural pouch lavage, aspiration of submandibular lymph node abscesses, milk, cutaneous swabs and uterine swabs [16]. In the present study, out of 121 horses with clinical signs sampled as nasopharyngeal swabs, the results of PCR included one of *Streptococcus dysgalactiae subsp. equisimilis*, 88 *S. zooepidemicus* and one *S. equi.*

Increasing the accuracy of diagnosis and reducing the detection time and differentiation of beta-hemolytic streptococci is an important step in controlling the prevalence of beta-hemolytic infections. PCR testing is also very sensitive for faster and more accurate detection of *S. equi* in horses with clinical signs compared to phenotypic methods [11]. The present study results further confirmed that out of 121 samples tested, 90 samples were positive for beta-hemolytic streptococci using PCR and only 70 samples were positive for bacterial culture and phenotypic methods.

In the present study, *S. zooepidemicus*, *S. equi* and *Streptococcus dysgalactiae subsp. equisimilis* were isolated from clinical specimens by PCR test (72.74%, 0.82% and 0.82%, respectively). In a similar study, *S. zooepidemicus*, *S. equi* and *Streptococcus dysgalactiae subsp. equisimilis* were isolated (72%, 21.3% and 5.8%, respectively) [14]. Also, in another study, the rate of *S. zooepidemicus* was 80.4%, *S. equi* 14.1% and *Streptococcus dysgalactiae subsp. equisimilis* 5.5% [17]. The results of these two studies showed that the isolation rate of these organisms was similar to the present study and the highest isolation rate in both studies was the same as *S. zooepidemicus* in the current study.

In one study, strangles-like disease and the prevalence of *S. zooepidemicus* was evaluated by RT PCR. In this study, IDEXX’s RT PCR based on strangles to screen all three strains of beta-hemolytic streptococci was used. The relevant results confirmed *S. zooepidemicus* showed that beta-hemolytic streptococci were better evaluated by IDEXX RT PCR and identified such outbreaks [18]. Other studies have shown that the absence of *S. equi* and the repeated identification of *Streptococcus dysgalactiae subsp. equisimilis* and *S. zooepidemicus* indicate that beta-hemolytic streptococci other than *S. equi* may be strangles-like [19]. The present study also showed that the main cause of strangles-like disease was in areas with seasonal prevalence of *S. zooepidemicus*, of which 88 cases were isolated from 121 horses.

PCR is a more sensitive method for detecting beta-hemolytic streptococci on nasopharyngeal swabs than culture. According to Newton et al. (2000), out of 61 positive nasopharyngeal swabs, the results of PCR test were 92% versus bacterial culture test of 30% [20]. Earlier research has primarily concentrated on the primer design within a genome region housing the superantigenic codes for *SeeH*, *SeeI*, *SeeL*, and *SeeM* toxins. According to research, Cordoni et al. (2015) used PCR‌ and RT-PCR for rapid diagnosis of strangles disease. These tests were very sensitive and used nasopharyngeal swabs, which facilitated the diagnosis of the organism on the same day [8]. This study utilizes the genomes of *SeM*, *SeeI* (a superantigenic component of toxins), *sodA*, and streptokinase for the identification of beta-hemolytic streptococci. In this study, similar to the mentioned studies, the results obtained from PCR test were 74% versus bacterial and phenotypic culture test, 57%.

A serial study was performed on *S.equi* isolates in carriers. 10 continuous carriers, of which 8 culture samples tested positive for *Streptococcus* were selected for this study. Of the 115 samples collected, 61 were qPCR positive. Of these, 32 were positive for culture. *SeM* sequences were determined for 6 of the 29 samples that were only qPCR positive. The prevalence of *S. equi* strain was *SeM*-72 [21]. In the present study, which lasted 4 months, a positive case of *S. equi* was isolated from 121 samples in PCR and sequencing was performed and the 97 *SeM* allele type was determined. The new allele of *S. equi SeM* in Iran was similar to the *SeM*-97 strain from England registered in the mentioned site [11]. In this study, we identified four amino acid alterations, specifically affecting aspartic acid, threonine, proline, and methionine, in the newly discovered *SeM*-97 allele when compared to *SeM*-3 of the reference strain 4047 of *S. equi*.

A study evaluated the relationship between age, race, and history of respiratory disease with high titers of anti-*SeM S. equi*-specific antibodies in serum. This study showed that factors such as sex, race and history of respiratory disease significantly increase the serum prevalence of carriers [22]. In contrast to the findings of our study, the results from the current investigation indicate that there isn’t a notable association between the occurrence of beta-hemolytic respiratory infections and gender or race variables. However, aligning with our study, a highly significant correlation was observed in terms of clinical signs, with horses displaying severe clinical symptoms testing positive for beta-hemolytic infections.

Also, in a study, the relationship between the history of respiratory disease and seroepidemiology of respiratory pathogens in Ethiopian working horses was investigated [23]. Similar to the present study’s findings (northwestern Iran), there was a significant relationship between the frequency of beta-hemolytic streptococci involving the respiratory tract with clinical signs recorded by a veterinarian and questioned by horse owners.

Strangles primarily involve younger horses, although the disease can occur at any age. Older horses often have an unusual and milder form of strangles disease, which is probably the result of cross-immunity from previous exposure to different strains of *S. equi* and *S. zooepidemicus*. Equestrian clubs that do not follow the principles of health and biosecurity and do not use separate equipment for each horse, increase the incidence of infections caused by beta-hemolytic streptococci involving the respiratory system at any age in horses [24, 25]. In the present study, in northwestern Iran, a significant relationship was observed between age and the prevalence of beta-hemolytic infections involving the respiratory system. Also, fewer positive cases were reported from older horses (over 10 years old), a sign of higher immunity and previous exposure to the disease. Horses exhibiting clinical indicators such as purulent nasal discharge, warmth, swelling, lymph node abscesses in the submandibular region and pharynx, as well as fever, tested positive for beta-hemolytic streptococci. All of these symptoms are typical signs of infections caused by beta-hemolytic streptococci involving the respiratory tract [10]. However, some symptoms including mucous-purulent discharge and abnormal respiratory sounds in hearing were also observed in horses with negative results of PCR (Table 6).

In Italy, a study by Laus et al. examined the prevalence of strangle-like disease clinically, endoscopically, cytologically, through bacterial culture and PCR. Of 28 horses, 30.8% had clinical signs of upper airway infection, including depression, purulent discharge, cough, and enlarged lymph nodes were reported, while 69.23% were clinically healthy [19]. In our current investigation, every horse displaying severe clinical symptoms (including warmth, swelling, abscesses in the submandibular and posterior retropharyngeal lymph nodes, and fever) yielded a positive outcome in beta-hemolytic streptococcal infections.

No studies have been performed on upper respiratory tract infections of beta-hemolytic streptococci origin in Iran. Our study, conducted in Iran, represents the pioneering effort in this particular domain, as previous research in the country has primarily focused on strangles disease through serological examinations and *S. equi* carriers.

In the current study, *S. zooepidemicus* (72.74%) was the most common factor isolated from the respiratory tract in adults and foals, consistent with a retrospective study conducted in the United States [14].

According to research on antibiotic resistance in bacteria isolated from horses between 1999 and 2012, 10 cases of *Streptococcus* isolated from horses in the South of England between 2007 and 2012 were resistant to antibiotics including: Enrofloxacin 40%, gentamicin 80%, penicillin or ceftiofur 0%, sulfonamides with trimethoprim 20%, doxycycline 10% and oxytetracycline 0%. Between 1999 and 2004, studies found that the utilization of enrofloxacin stood at 5.9%, while gentamicin usage was at 35.3%, signifying antibiotic overutilization and a corresponding rise in drug resistance [26]. In the present study, antibiotic resistance in streptococcal isolates from northwestern Iran was as follows: enrofloxacin 27.8%, gentamicin 67.8%, penicillin 7.8%, sulfamethoxazole with trimethoprim 47.8%, tetracycline 2/22%. High resistance in the present study is due to the indiscriminate use of antibiotics in the region.

## Conclusion

Strangles is an important infectious disease that has a major impact on the horse breeding clubs. Buying and selling horses and moving them around the world is important, so it is important to know more about the species in different countries and to identify their genetic relationship with each other to improve control and understanding of the disease [27, 28]. In our study, an *S. equi* genotype was identified. The 97 allele of this gene has not been officially reported anywhere and is only registered in the PubMLST-*SeM* database by Katy Webb. This was the first isolate reported and registered in the mentioned database. The Tabriz61 isolate exhibited the *SeM-97* allele, accompanied by clinical manifestations such as mucopurulent discharge, unusual lung sounds, warmth, swelling, or discharge in the retropharyngeal lymph node, and the presence of fever. This isolate was sensitive to penicillin, meropenem, ampicillin, cefotaxime, tetracycline, erythromycin, azithromycin, chloramphenicol, enrofloxacin and ciprofloxacin antibiotics and resistant to trimethoprim-sulfamethoxazole and gentamicin antibiotics. Because until the time of this study, no study had been done in Iran on *SeM* gene sequencing, this is the first report of the presence of this gene in the region. Hence, it is suggested that studies in different areas in Iran monitor this gene better when an outbreak occurs.

## Materials and methods

### Sample collection

The type of study was observational and cross-sectional analytical study. The study population was horses with clinical signs and the relationship between these horses with variables of age, sex, breed, clinical signs, and the geographical area was evaluated. Managers of some equestrian clubs in the northwestern provinces of Iran (West Azerbaijan, East Azerbaijan and Ardabil) were questioned regarding the occurrence of any respiratory disease or suspected disease cases in the above equestrian areas. As soon as they were informed of the disease, they went to the club and the infected horses were clinically evaluated. After clinical confirmation of respiratory involvement in the examined horses, all clinical signs and characteristics of the studied horses were recorded individually in the designed worksheets. Clinical signs in the studied areas including: fever, respiratory distress, presence of nasal secretions (mucous-purulent), warmth, swelling of lymph nodes in the head were recorded in special worksheets. Between October 2020 and January 2021, a total of 121 horses, spanning from 1 to 21 years of age and representing diverse breeds such as cross-breed, Arabian, Kurdish, and Thoroughbred were sampled due to the presence of clinical symptoms. Sampling was done by using a designed nasopharyngeal swab (60 cm for adults and 30 cm for foals) from horses with clinical signs of respiratory diseases from 18 horse breeding clubs. The procedure was performed in such a way that the swabs were passed through the nasal canal, and after stimulating the animal to perform several swallowing operations to release the secretions from the pharyngeal tube and ensure that the swabs were impregnated with the secretions, the swabs were taken out of the nasal canal. Swabs were transferred to a bacterial laboratory in Amies agar gel transport medium.

### Bacterial culture

Biochemical staining, bacterial isolation, and subsequent biochemical analysis were conducted on the collected samples. The differentiation of all streptococcal isolates was achieved through a series of biochemical tests. These biochemical experiments included fermentation of trehalose, lactose, maltose, sorbitol, inulin, mannitol, raffinose, salicin, esculin hydrolysis, and sodium hippurate and growing in 6.5% NaCl [29].

### Preparation of DNA from cultured colonies

Isolates were stored in the laboratory after biochemical confirmation. For short-term storage for daily tests, linear infusion agar was linearly cultured and refrigerated. To prevent genetic changes, glycerin was stored at -70 °C for long-term storage. Pure and biochemically isolated isolates were used for DNA extraction. The gram-positive bacterial DNA extraction kit (MBST CO., Iran) was used. On the bacterial pellet, 100 µl of homogeneous buffer (HB) was added and after centrifugation, the supernatant was discarded. 150 µl HB buffer was poured back onto the pellet and vertex until uniform. 20 µl of lysozyme solution was added to the high uniform solution and incubated for 4 h at 37 °C. 180 µl of Lysis buffer solution was added and vortexed and incubated for 10 min at 55 °C. 360 µl of Binding buffer solution was added to the top microtube and vortexed for 30 s and incubated for 10 min at 70 °C by a Thermoblock device (Block Heater, 2 Block, Digital, SBH130D, UK). 270 µl of 100% ethanol alcohol was added and vortexed. The contents of the above microtubes were transferred to the columnar microtubes in the kit and centrifuged at 8000 g for one minute. The centrifuged solution was discarded and the DNA column was transferred to a new microtube. 500 µl of Wash buffer solution was added to the column and after centrifugation at 8000 g rpm for 1 min, the filtered solution was discarded and this step was repeated. The DNA column was placed in a new microtube and centrifuged at high speed for two minutes to remove the filter from any of the above solutions and to dry the DNA. The column was moved to sterile microtubes, followed by the addition of 100 µl of Elution buffer solution. The mixture was allowed to sit at room temperature for 10 min before being centrifuged at 8000 g. Subsequently, the resulting solution underwent assessment for both DNA quality and quantity using nanodrop and agarose gel analysis. The DNAs were stored at -20 °C for further research. After extraction by kit, all DNAs were concentrated by a photometer (BioRad, USA) and their values were checked at OD 260/280 and OD 280/230, which were very suitable in terms of purity. Also, 5 µl of each DNA were taken on an agarose gel to be tested for quality, which was a good result.

### PCR of sodA, seeI, seM and streptokinase genes

This PCR was used to confirm all isolates identified as *S. equi, S. zooepidemicus* and *Streptococcus dysgalactiae subsp. equisimilis* by biochemical tests. For this purpose, all isolates were examined with a specific primer pair (Table 9) synthesized by Metabion (Germany). The bacterial 16 S rRNA gene was used to confirm the absence of PCR inhibitors. Furthermore, a positive strain of *S. zooepidemicus* (ATCC 35,195) was obtained from the Center for Genetic and Biological Resources of Iran, identified by the code IBRC-M No. 10,919 and *S. equi* strain prepared from the microbial collection of the Faculty of Veterinary Medicine, the University of Tehran with access code MZ292707 of NCBI database was used. The PCR product was prepared in a final volume of 25 µl using 12.5 µl of ready-made master mix (Catalogue No. MM2062, Sinaclon, Iran), 1 µl of each primer (10 picomol concentration), 3 µl of DNA and 7.5 µl of distilled water. The first step of PCR was the initial opening of the template DNA at 94 °C for 3 min. The next steps of PCR were performed in 35 cycles in the following order: 94 °C for one minute to open the template DNA strands, temperature 55 or 59 °C according to Table 9 for one minute to connect the primers to the pattern strands, 72 °C for one minute to polymerize the new strand from the pattern strand. In the final cycle, a temperature of 72 °C was used for 10 min to complete the polymerization of incomplete filaments. To verify the size of the specific bands, we also employed a 100 bp Ladder (Catalog No. SL7041, sourced from Sinaclon, Iran). The PCR procedures were carried out using a T100 Thermocycler (T100, provided by BioRad, USA). 1.5% agarose gel was electrophoresed with 85 volts for 90 min in 1X TBE buffer to read the PCR results [30].

**Table 9:** Oligonucleotide primers used in PCR to confirm the diagnosis of beta-hemolytic streptococci

Primers	Nucleotide sequences	Target gene	Size (bp)
16S_F 16S_R	5’-AGAGTTTGATCMTGGCTCAG-3’ 5’-GCTGCCTCCCGTAGGAGT-3’	*16 S rRNA*	354
SeM_F SeM_R	5’-CAGAAAACTAAGTGCCGGTG-3’ 5’-ATTCGGTAAGAGCTTGACGC-3’	*SeM*	541
sodA_F sodA_R	5’-CAGCATTCCTGCTGACATTCGTCAGG-3’ 5’-CTGACCAGCATTATTCACAACCAGCC-3’	*sodA*	235
Kin_F Kin_R	5’-TCAAATCGGTTGGCACAGAC-3’ 5’-CGTCCTTAGCATAGAAGGATTGG-3’	*Streptokinase*	279
SeeI_F SeeI_R	5’-GAAGGTCCGCCATTTTCAGGTAGTTTG-3’ 5’-GCATACTCTCTCTGTCACCATGTCCTG-3’	*SeeI*	520

### Sequence analysis

Isolates DNA (one case of *S. equi*, one case of *Streptococcus dysgalactiae subsp. equisimilis* and 11 cases of *S. zooepidemicus*) were sent to Codon Genetics for partial genome sequencing. The reason for selecting these 11 *S. zooepidemicus* isolates for partial genome analysis was that these isolates caused more severe clinical symptoms in horses. Sequences of *SeM*, *SeeI*, *sodA* and *streptokinase* genes were analyzed by Mega 7 software. Forward and reverse sequences are then agreed upon by the PRABI-Doua site. The GeneMarks program on the GenSAS site analyzed the integrated FASTA file in more detail. These sequences were recorded by the Bankit program at the NCBI database and an access number was assigned. The allele of the *SeM* gene was identified in the isolated isolate by comparing the sequences of this gene in the NCBI database (https://pubmlst.org/szooepidemicus/seM) [30]. Cloudograms of *SeM* and *sodA* genes were plotted using the Neighbor-joining method with Kimura 2 Parameter statistical model [31] and BootStrap 1000 by Mega 7 software [32].

### Determination of antibiotic susceptibility and resistance

To determine the antibiotic susceptibility of the isolates, the disk diffusion method (Kirby-Bauer method) was used according to the recommended CLSI guidelines for 2020 [33]. Using a sterile swab, the bacteria were removed from a liquid medium equal to a concentration of half McFarland and cultured uniformly on a Müller-Hinton medium enriched with 5% sheep blood and prepared on a plate, in different directions. Then, within 15 min after culture, antibiotic disks were placed on the surface of the medium immediately using forceps and the final incubation was performed for 48 to 72 h at 37 °C. To determine the susceptibility, 12 antibiotic discs of MAST Company (UK) were used, including disks cefotaxime (CTX 30 µg), tetracycline (T 30 µg), trimethoprim-sulfamethoxazole (TS 25 µg), meropenem (MEM 10 µg), chloramphenicol (C 30 µg), erythromycin (E 30 µg), azithromycin (ATH 15 µg), ciprofloxacin (CIP 5 µg), penicillin (P 10 µg), gentamicin (G 10 µg), ampicillin (AP 10 µg) and enrofloxacin (ENF 5 µg). Finally, a caliper measured the growth inhibition area created around each disk in millimeters. By measuring the diameter of the halo, sensitivity and comparison with standard tables, resistance (R), intermediate (I) and sensitivity (S) to antibiotics were evaluated. Standard sizes were used according to the manufacturer’s instructions to interpret the results.

### Data analysis

SPSS software version 26 was used to analyze phenotypic, molecular and antibiotic susceptibility data and descriptive methods (calculation of absolute and relative frequency) and chi-square test was used to compare the results obtained in this study and in all stages *P* < 0.05 was considered significant.

### Electronic supplementary material

Below is the link to the electronic supplementary material.

Supplementary Material 1

## Data Availability

All the data supporting the findings of this study are included in the article. The data that support the findings of this study are openly available in GenBank database at https://www.ncbi.nlm.nih.gov/genbank/ (GenBank accession number OL332313, OL332314, OK554432, OK626590 and OK663653 to OK663661).

## List of abbreviations

PubMLST: Public databases for molecular typing and microbial genome diversity

## References

[1] Sellon DC, Long MT. Equine infectious diseases E-book. Elsevier Health Sciences; 2013.

[2] Timoney JF (2004). The pathogenic equine streptococci. Vet Res.

[3] Constable PD, Hinchcliff KW, Done SH, Grünberg W. Veterinary medicine: a textbook of the diseases of cattle, horses, sheep, pigs and goats. Elsevier Health Sciences; 2016.

[4] Taylor SD, Wilson WD (2006). Streptococcus equi subsp. equi (strangles) infection. Clin Techniques Equine Pract.

[5] Paillot R, Lopez-Alvarez M, Newton J, Waller A, Strangles. A modern clinical view from the 17th century. Wiley Online Library; 2017. pp. 141–5. 10.1111/evj.12659 28177153

[6] Timoney JF, Strangles (1993). Veterinary Clin North America: Equine Pract.

[7] Chanter N (1997). Streptococci and enterococci as animal pathogens. J Appl Microbiol.

[8] Cordoni G, Williams A, Durham A, Florio D, Zanoni RG, La Ragione RM (2015). Rapid diagnosis of strangles (Streptococcus equi subspecies equi) using PCR. Res Vet Sci.

[9] Delph KM, Beard LA, Trimble AC, Sutter ME, Timoney JF, Morrow JK (2019). Strangles, convalescent Streptococcus equi subspecies equi M antibody titers, and presence of complications. J Vet Intern Med.

[10] Sweeney CR, Timoney JF, Newton JR, Hines MT (2005). Streptococcus equi infections in horses: guidelines for treatment, control, and prevention of strangles. J Vet Intern Med.

[11] Webb K, Barker C, Harrison T, Heather Z, Steward KF, Robinson C (2013). Detection of Streptococcus equi subspecies equi using a triplex qPCR assay. Vet J.

[12] Boyle A (2017). Strangles and its complications. Equine Veterinary Education.

[13] Clark C, Greenwood S, Boison JO, Chirino-Trejo M, Dowling PM (2008). Bacterial isolates from equine infections in western Canada (1998–2003). Can Veterinary J.

[14] Erol E, Locke SJ, Donahoe JK, Mackin MA, Carter CN (2012). Beta-hemolytic Streptococcus spp. from horses: a retrospective study (2000–2010). J Vet Diagn Invest.

[15] Panchaud Y, Gerber V, Rossano A, Perreten V (2010). Bacterial infections in horses: a retrospective study at the University equine clinic of Bern. Schweiz Arch Tierheilkd.

[16] Preziuso S, Cuteri V (2012). A multiplex polymerase chain reaction assay for direct detection and differentiation of β-hemolytic streptococci in clinical samples from horses. J Equine Veterinary Sci.

[17] Alper M. Isolation and antibiotic susceptibilities of beta hemolytic Streptococcus species from various body site infections with cytologic evidences in Thoroughbred and Arabian racehorses in Turkey. Kafkas Üniversitesi Veteriner Fakültesi Dergisi.24(1).

[18] Vin VR, Bishop I, Leutenegger C (2016). Strangles-like disease and outbreaks caused by Streptococcus equi subspecies zooepidemicus: case cluster description and diagnostics by a real-time PCR strangles screen. J Equine Veterinary Sci.

[19] Laus F, Preziuso S, Spaterna A, Beribe F, Tesei B, Cuteri V (2007). Clinical and epidemiological investigation of chronic upper respiratory diseases caused by beta-haemolytic streptococci in horses. Comp Immunol Microbiol Infect Dis.

[20] Newton J, Verheyen K, Talbot N, Timoney J, Wood J, Lakhani K (2000). Control of strangles outbreaks by isolation of guttural pouch carriers identified using PCR and culture of Streptococcus equi. Equine Vet J.

[21] Riihimäki M, Aspán A, Ljung H, Pringle J (2018). Long term dynamics of a Streptococcus equi ssp equi outbreak, assessed by qPCR and culture and seM sequencing in silent carriers of strangles. Vet Microbiol.

[22] Boyle AG, Sweeney CR, Kristula M, Boston R, Smith G (2009). Factors associated with likelihood of horses having a high serum Streptococcus equi SeM-specific antibody titer. J Am Vet Med Assoc.

[23] Laing G, Christley R, Stringer A, Aklilu N, Ashine T, Newton R (2018). Respiratory disease and sero-epidemiology of respiratory pathogens in the working horses of Ethiopia. Equine Vet J.

[24] Duran MC, Goehring LS (2021). Equine strangles: an update on disease control and prevention. Austral J Veterinary Sci.

[25] Waller AS (2014). New perspectives for the diagnosis, control, treatment, and prevention of strangles in horses. Veterinary Clinics: Equine Practice.

[26] Johns I, Adams EL (2015). Trends in antimicrobial resistance in equine bacterial isolates: 1999–2012. Vet Rec.

[27] Ivens P, Matthews D, Webb K, Newton J, Steward K, Waller A (2011). Molecular characterisation of ‘strangles’ outbreaks in the UK: the use of M-protein typing of Streptococcus equi ssp. equi. Equine Vet J.

[28] Libardoni F, Vielmo A, Farias L, Matter LB, Pötter L, Spilki FR (2013). Diversity of seM in Streptococcus equi subsp. equi isolated from strangles outbreaks. Vet Microbiol.

[29] Quinn PJ, Markey BK, Leonard FC, Hartigan P, Fanning S, Fitzpatrick E. Veterinary microbiology and microbial disease. John Wiley & Sons; 2011.

[30] Kelly C, Bugg M, Robinson C, Mitchell Z, Davis-Poynter N, Newton JR (2006). Sequence variation of the SeM gene of Streptococcus equi allows discrimination of the source of strangles outbreaks. J Clin Microbiol.

[31] Saitou N, Nei M (1987). The neighbor-joining method: a new method for reconstructing phylogenetic trees. Mol Biol Evol.

[32] Kumar S, Stecher G, Tamura K (2016). MEGA7: molecular evolutionary genetics analysis version 7.0 for bigger datasets. Mol Biol Evol.

[33] Wayne P. Performance standards for antimicrobial susceptibility testing; 30th informational supplement. CLSI document M100-S30. Clinical and Laboratory Standards Institute; 2020.

