# Supplementary material for: Molecular and sequencing study and identification of novel SeM-type in beta-hemolytic streptococci involving the upper respiratory tract in Iran
Source: BMC Vet Res. 2023 Oct 17;19:210. doi: 10.1186/s12917-023-03772-4 (PMC10580511; doi:10.1186/s12917-023-03772-4)
Supplement: Supplementary file 1 — Supplementary Material 1 [file 12917_2023_3772_MOESM1_ESM.pdf]

Supplementary Info File:

Figure S1

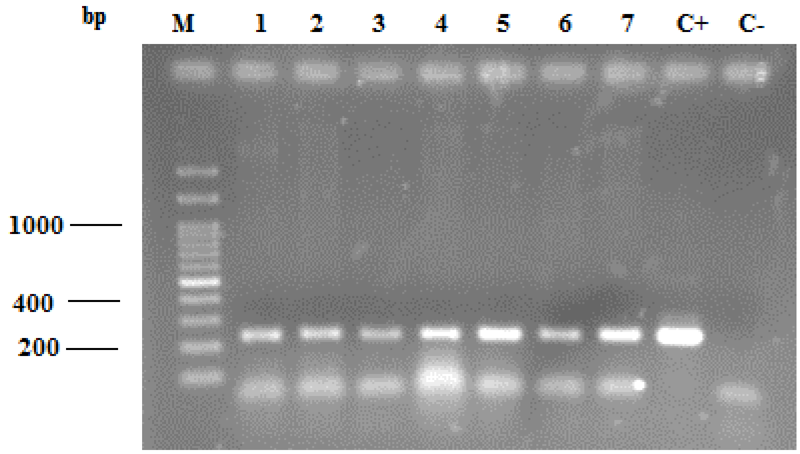

Figure S2

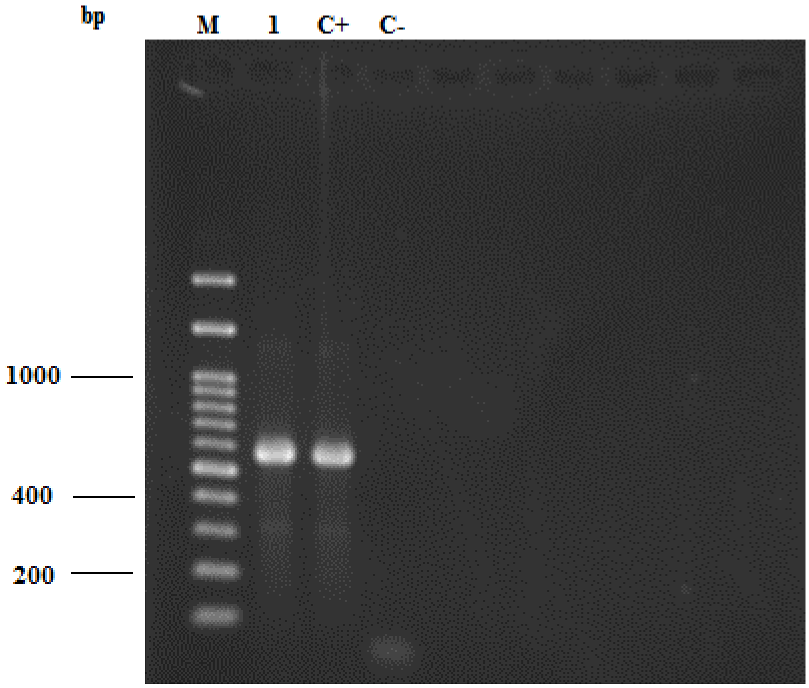

Figure S3

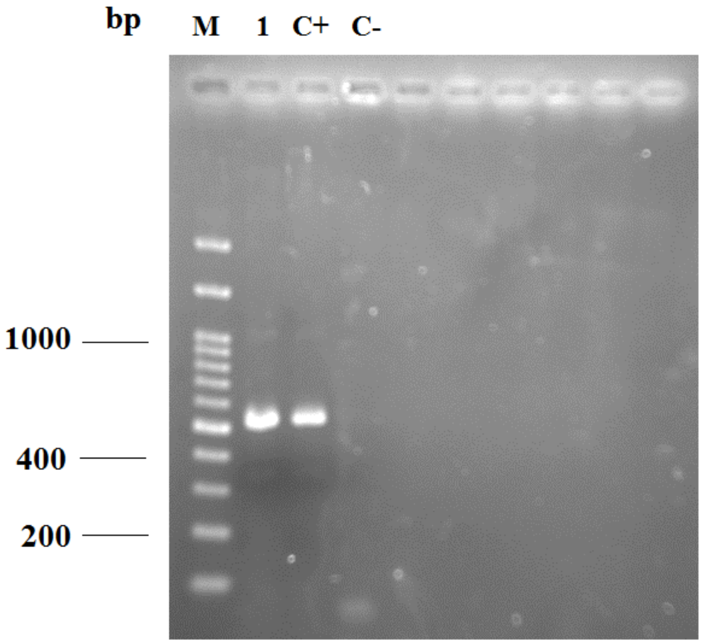

Figure S4

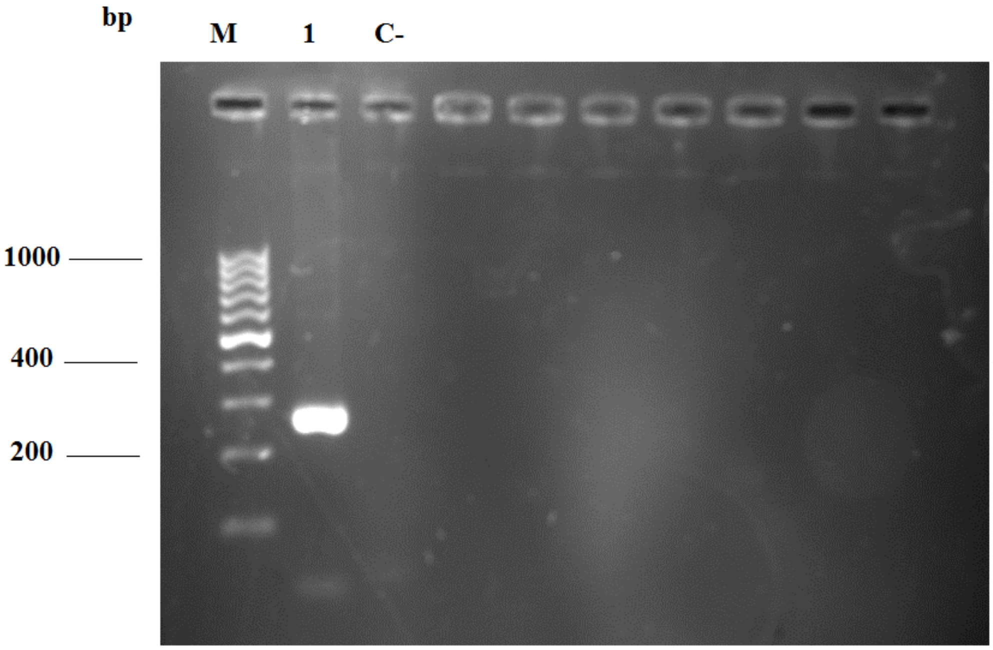

Figure S5

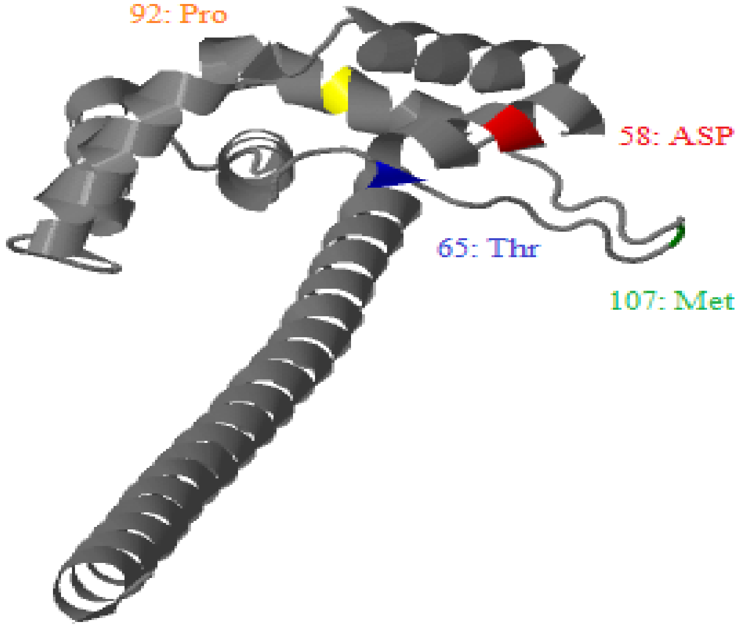

Figure Legends

Figure S1: Electrophoresis of the final PCR product on the *sodA* gene in a number of *Streptococcus zooepidemicus* isolates

M: Marker 100bp, Lane 1 to 7: Some *Streptococcus zooepidemicus* isolates, C+ (Positive control): *Streptococcus zooepidemicus* ATCC 35195, C-: Negative control

Figure S2: Electrophoresis of the final PCR product on the *SeM* gene in a number of *Streptococcus equi* isolate

M: Marker 100bp, Lane 1: *Streptococcus equi* isolate, C+ (Positive control): *Streptococcus equi* MZ292707, C-: Negative control

Figure S3: Electrophoresis of the final PCR product on the *SeeI* gene (OK663661) in a number of *Streptococcus equi* isolate

M: Marker 100bp, Lane 1: *Streptococcus equi* isolate, C+ (Positive control): *Streptococcus equi* prepared from microbial collection, C-: Negative control

Figure S4: Electrophoresis of the final PCR product on the *Streptokinase* gene in a number of *Streptococcus dysgalactiae subsp. equisimilis* isolate

M: Marker 100bp, Lane 1: *Streptococcus dysgalactiae subsp. equisimilis* isolate, C-: Negative control

Figure S5: Three-dimensional model of protein gene *SeM-97*
